# Expression Profiles of Hypoxia-Related Genes of Cancers Originating from Anatomically Similar Locations Using TCGA Database Analysis

**DOI:** 10.3390/medicines11010002

**Published:** 2023-12-31

**Authors:** Hye Lim Bae, Kyeonghun Jeong, Suna Yang, Hyeji Jun, Kwangsoo Kim, Young Jun Chai

**Affiliations:** 1 Department of Surgery, Seoul National University College of Medicine, Seoul 03080, Republic of Korea; gpfla1206@gmail.com; 2 Interdisciplinary Program in Bioengineering, Seoul National University, Seoul 08826, Republic of Korea; scientist0205@snu.ac.kr; 3 Department of Clinical Medical Science, Seoul National University, Seoul 08826, Republic of Korea; tjsdk3105@gmail.com; 4 Seoul National University Hospital Biomedical Research Institute, Seoul 03080, Republic of Korea; hjjshine@gmail.com; 5 Department of Transdisciplinary Department of Medicine, Institute of Convergence Medicine with Innovative Technology, Seoul National University Hospital, Seoul 03080, Republic of Korea; 6 Department of Medicine, Seoul National University College of Medicine, Seoul 03080, Republic of Korea; 7 Department of Surgery, Seoul Metropolitan Government—Seoul National University Boramae Medical Center, Seoul 07061, Republic of Korea

**Keywords:** bioinformatics, hypoxia, genomics, amino acids

## Abstract

**Background:** Hypoxia is a well-recognized characteristic of the tumor microenvironment of solid cancers. This study aimed to analyze hypoxia-related genes shared by groups based on tumor location. **Methods:** A total of 9 hypoxia-related pathways from the Kyoto Encyclopedia of Genes and Genomes database or the Reactome database were selected, and 850 hypoxia-related genes were analyzed. Based on their anatomical locations, 14 tumor types were categorized into 6 groups. The group-specific genetic risk score was classified as high- or low-risk based on mRNA expression, and survival outcomes were evaluated. **Results:** The risk scores in the Female Reproductive group and the Lung group were internally and externally validated. In the Female Reproductive group, CDKN2A, FN1, and ITGA5 were identified as hub genes associated with poor prognosis, while IL2RB and LEF1 were associated with favorable prognosis. In the Lung group, ITGB1 and LDHA were associated with poor prognosis, and GLS2 was associated with favorable prognosis. Functional enrichment analysis showed that the Female Reproductive group was enriched in relation to cilia and skin, while the Lung group was enriched in relation to cytokines and defense. **Conclusions:** This analysis may lead to better understanding of the mechanisms of cancer progression and facilitate establishing new biomarkers for prognosis prediction.

## 1. Introduction

Hypoxia is a common feature of the tumor microenvironment of malignant solid tumors that promotes invasive and metastatic tumor behaviors, and activates expression of various hypoxia-related genes such as the hypoxia-inducible factor [1]. Hypoxic foci are formed when cancer cells’ metabolic requirement surpasses the intravascular oxygen available. By inducing epithelial-to-mesenchymal transition, hypoxic microenvironments are associated with poor outcomes and reduced survival [2]. Several hypoxia-related genes are biomarkers for the prognosis prediction of common malignancies such as breast, colorectal, gastric, and thyroid cancers [3,4].

Cancers originating in anatomically close organs may have similar genetic profiles. For example, the mutational profiles of distal colon and rectal cancer are similar, although the two cancers display distinct clinical behaviors [5]. Likewise, esophageal and gastric adenocarcinomas share commonalities in targeted therapeutic strategies and clinical outcomes, particularly in terms of mortality rates [6]. They also have similar mutational rates in the *APC*, *KRAS*, *PTEN*, and *SMAD4* genes [7]. In the context of urinary tract malignancies, previous studies have demonstrated the similarity in the gene expression profiles of urothelial carcinomas originating from both the upper tract (ureter and renal pelvis) and the lower tract (bladder and urethra) [8]. These findings suggest the existence of comparable pathogenic mechanisms governing the development of the tumors. Among the various cancer mechanisms, hypoxia is commonly involved in tumor progression, and cancers originating in organs situated close to each other may have similar expression profiles of hypoxia-related genes.

The Cancer Genome Atlas (TCGA) database provides multiplatform genomic data of more than 20 types of carcinomas [9]. It includes data about microsatellite instability, DNA sequencing, miRNA sequencing, protein expression, mRNA sequencing, DNA methylation, copy number variation, clinical information, and clinical images. The TCGA project produced genomic data under standardized and controlled conditions, making it an ideal platform for pan-cancer analyses.

In this study, we hypothesized the involvement of specific hypoxia-related genes that exhibit commonality across cancers originating from anatomically similar locations. To investigate this hypothesis, we utilized the TCGA database to categorize 14 different cancer types into 6 groups based on their anatomical origin, and analyzed the group-specific genetic risk scores with mRNA expression levels. Subsequently, we sought to identify the genes with the potential to predict survival outcomes through the analysis of gene expression profiles associated with hypoxia.

## 2. Materials and Methods

### 2.1. Selection of Hypoxia-Related Genes

We compiled the hypoxia-related gene list by searching through the literature regarding pan-cancer samples (Table S1), and gene ontology analysis was performed using data from the Kyoto Encyclopedia of Genes (KEGG) and the Reactome database to find the hypoxia-related gene pathways. Among the pathways, we manually selected 9 pathways that hold clinical significance (Table 1). Then, we identified 850 genes that are involved in the 9 hypoxia-related pathways.

Fourteen tumor types in the TCGA were categorized into six groups based on their anatomical locations, as shown in Table 2. The Liver group comprised liver hepatocellular carcinoma and cholangiocarcinoma; the Upper Gastrointestinal (GI) group comprised esophageal carcinoma and stomach adenocarcinoma; the Lower GI group comprised colon adenocarcinoma and rectum adenocarcinoma; the Female Reproductive group comprised uterine corpus endometrial carcinoma, cervical squamous cell carcinoma, and endocervical adenocarcinoma; the Urinary group comprised bladder urothelial carcinoma, kidney renal clear cell carcinoma, kidney renal papillary cell carcinoma, and kidney chromophobe; and the Lung group comprised lung adenocarcinoma and lung squamous cell carcinoma (Table 2).

The TCGA pan-cancer RNA-seq data and the TCGA clinical data outcome resource were downloaded from the PanCanAtlas publications page (accessed on 18 December 2020, https://gdc.cancer.gov/about-data/publications/pancanatlas). A total of 4751 tumor samples with TCGA-CDR outcome data were used for downstream analysis [10]. Among the 850 hypoxia-related genes, 6 genes (*LOC101928143*, *LOC102723407*, *MIR1281*, *PLA2G4B*, *SLC5A10*, and *G6PC1*) were excluded from the TCGA pan-cancer RNA-seq data; thus, 844 genes with RNA expression values were used in the analysis.

### 2.2. Group-Specific Genetic Risk Gene Identification

We compiled data encompassing all genes associated with hypoxia and then proceeded to perform Cox regression analyses to compute individualized genetic risk scores for each of the specified groups. Elastic net penalized Cox regression analysis was conducted based on the RNA expression values of 844 hypoxia-related genes and each group’s overall survival data. Genes with Cox regression model coefficients that were not equal to 0 were defined as group-specific genetic risk genes. The group-specific genetic risk score, Rg, was calculated using the following equation:

$$Genetic\;risk\;score,R_{g}=\sum_{i=1}^{n}b_{g,i}x_{g,i}$$

where g represents group classification according to anatomical location, i represents the number of genes with coefficients not equal to 0, b represents the elastic net Cox regression coefficient value of each gene, and x represents the RNA expression value of each gene. The Glmnet R package was employed for elastic net Cox regression analysis [9].

### 2.3. Survival Analysis and Internal Validation

Group samples were classified as ‘high-risk’ or ‘low-risk’ based on the median value of the group-specific genetic risk score. Survival analysis was conducted using Kaplan–Meier survival curves. Log-rank tests were performed to compare survival curves between high-risk and low-risk groups. Tumor types with log-rank test *p*-values between high-risk and low-risk classes below 0.05 across all tumor types within each group were defined as internally validated groups. The R package *pROC* was used for building receiver operating characteristic (ROC) curves and *survminer* was used for the Kaplan–Meier plot and log-rank test [11].

### 2.4. External Validation

To improve the validity and generalizability, external validation was performed on the internally validated groups, using the dataset of the Gene Expression Omnibus (GEO). Nineteen external validation datasets were obtained from the GEO database (accessed on 1 January 2022, https://www.ncbi.nlm.nih.gov/geo/, Table S1). Log2-transformed raw expression values were used for risk score calculation. For each dataset, samples were classified as high- and low-risk by their calculated genetic risk score. We calculated the gene coefficients of the groups (Table 3). R packages *GEOquery*, *Affy*, *Oligo*, and *Limma* were used for the pre-processing of external validation datasets. To detect and remove outliers, the *outlier_osd* function of the R package *survBootOutliers* was applied. Survival analysis was performed by dividing samples of each dataset into high- and low-risk classes based on the median genetic risk score.

### 2.5. Functional Enrichment Analysis

Gene set enrichment analysis (GSEA) was conducted using the R package clusterProfiler [12]. KEGG pathway and Gene Ontology Biological Process terms from the Molecular Signatures Database were used to perform functional annotation of differentially expressed genes (DEGs) between high- and low-risk classes within each validated group. The top ten enriched terms were extracted in this study. Among the genes with an average of more than three RNA expression values, DEGs between high- and low-risk classes within each group were identified with a cutoff value at Wilcoxon rank-sum test false discovery rate < 0.01 and absolute log2 fold change > 1.

### 2.6. Gene Ontology Analysis

Gene ontology analysis was conducted using the R package *clusterProfiler* [12]. KEGG pathway and Gene Ontology biological process terms from the Molecular Signatures Database were used to perform functional annotation of positive risk coefficient genes within each validated group. The 844 hypoxia-related genes were used as a background gene set. The top 10 terms among enriched terms with a q-value of less than 0.2 were represented as dot plots.

## 3. Results

### 3.1. Selection of Hypoxia-Related Genes

From the hypoxia-related pathways, 850 genes involved in hypoxia-related pathways were selected and defined as hypoxia-related genes. Among the 850 hypoxia-related genes, 6 genes (*LOC101928143*, *LOC102723407*, *MIR1281*, *PLA2G4B*, *SLC5A10*, and *G6PC1*) were absent from the TCGA pan-cancer RNA-seq data; thus, 844 genes with RNA expression values were used in the analysis.

### 3.2. Group-Specific Genetic Risk Score Identification

**Figure 1:**
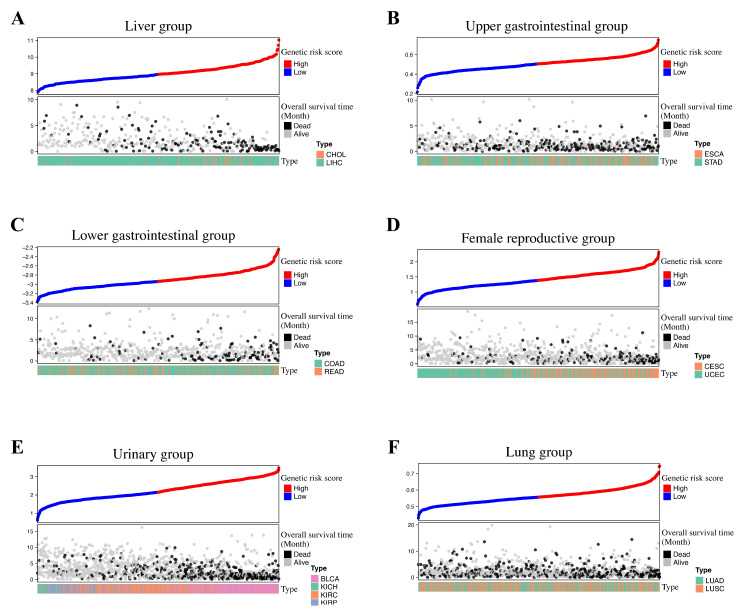
Risk score distribution plot of each group. The upper panels are ordered by genetic risk scores, categorized into high- (red dots) and low- (blue dots) risk based on the median. The lower panels represent overall survival time in months, with patient survival status denoted by black (deceased) and grey (alive) dots. In the Female Reproductive group, 79% of patients with the CESC tumor type were classified as high-risk. Risk scores were evenly distributed across tumor types in the Upper GI, Lower GI, and Lung groups. (**A**) Liver group, (**B**) Upper GI group, (**C**) Lower GI group, (**D**) Female Reproductive group, (**E**) Genitourinary group, (**F**) Lung group.

Group-specific genetic risk genes and coefficients are listed in Table 4 In accordance with the coefficient’s direction, we have compiled the results into a table that distinguishes positive and negative associations. The distribution of hypoxia genetic scores is shown in the risk score plot (Figure 1). In the Female Reproductive group, 232 (79%) out of the 293 patients with the CESC tumor type were classified as high-risk. Risk scores were evenly distributed across tumor types in the Upper GI, Lower GI, and Lung groups.

### 3.3. Survival Analysis and Internal Validation

**Figure 2:**
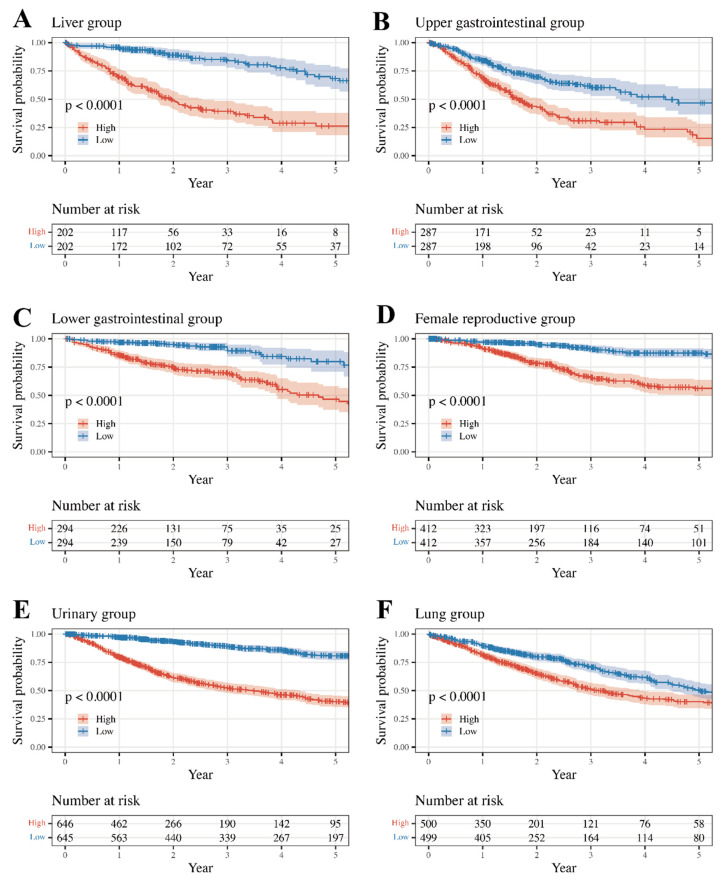
Comparison of Kaplan–Meier survival curves between high- and low-risk classes. (**A**) OS in Liver group, (**B**) OS in Upper GI group, (**C**) OS in Lower GI group, (**D**) OS in Female Reproductive group, (**E**) OS in Genitourinary group, (**F**) OS in Lung group; OS, overall survival; GI, Gastrointestinal.

**Figure 3:**
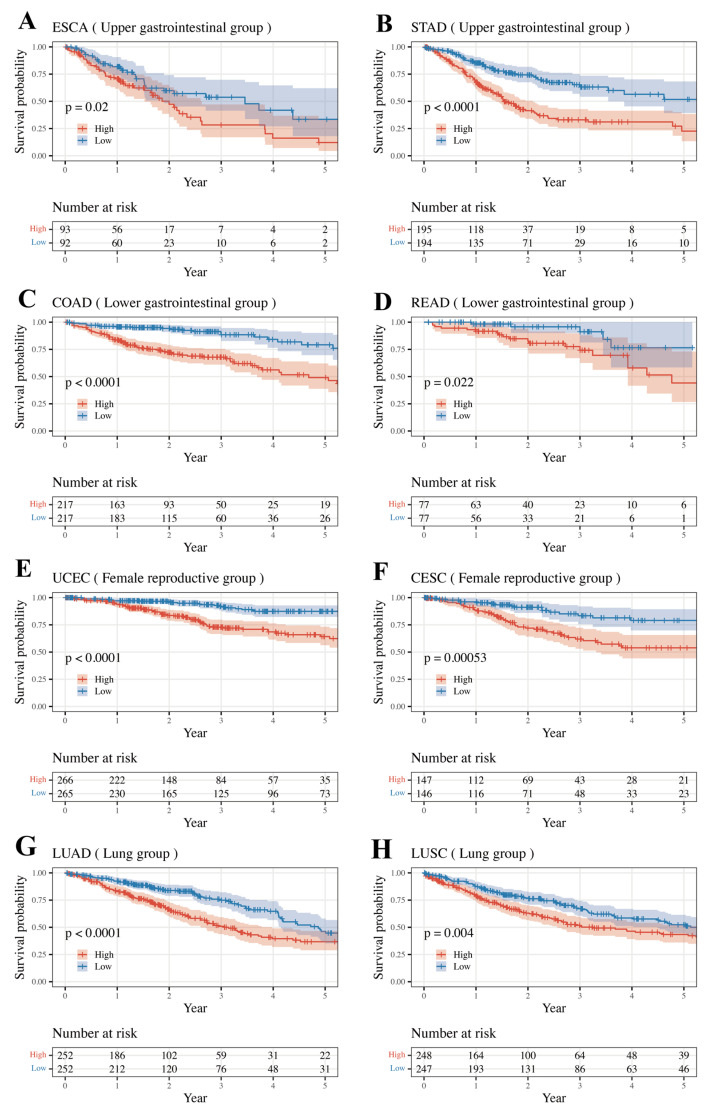
Comparison of Kaplan–Meier survival curves between high- and low-risk classes based on the median risk score. The Female Reproductive, Lung, Upper Gastrointestinal, and Lower Gastrointestinal groups were internally validated. (**A**) OS in ESCA, (**B**) OS in STAD, (**C**) OS in COAD, (**D**) OS in READ, (**E**) OS in UCEC, (**F**) OS in CESC, (**G**) OS in LUAD, (**H**) OS in LUSC; OS, overall survival.

Overall survival rates between high- and low-risk classes were compared using the Kaplan–Meier method and the log-rank test (Figure 2). To assess the risk score performance of each tumor, samples were divided into high- and low-risk classes based on the median risk score within each group, and survival was compared using the Kaplan–Meier method and the log-rank test. The Female Reproductive, Lung, Upper GI, and Lower GI groups were internally validated (Figure 3). The log-rank test p-values comparing the survival rates of the high- and low-risk classes are listed in Table 4. Except for CHOL and KICH, the remaining cancer types showed significantly different results. (*p*-value < 0.05)

### 3.4. External Validation Result of Internally Validated Groups

Risk scores were calculated using the group-specific genetic risk score gene coefficients calculated from TCGA samples (Table 5).

**Figure 4:**
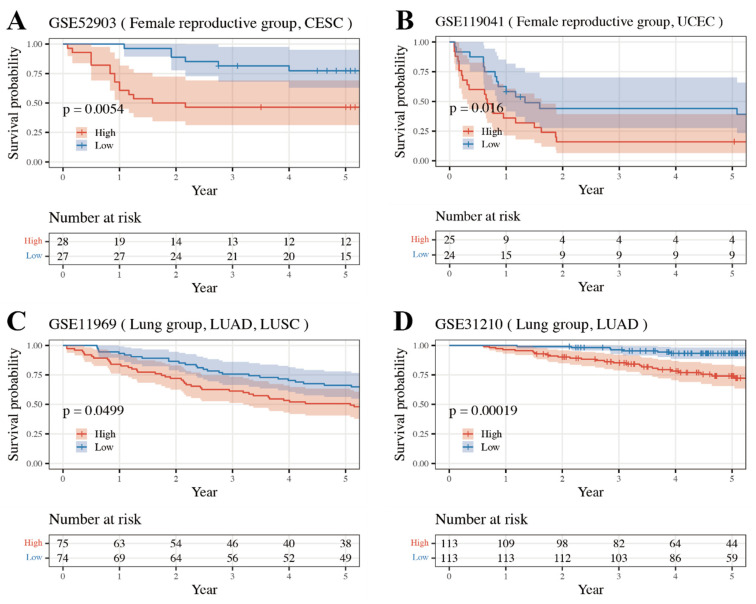
Kaplan–Meier plot of each external validation dataset. The Female Reproductive and Lung groups were externally validated. (**A**) OS in GSE52903, (**B**) OS in GSE119041, (**C**) OS in GSE11969, (**D**) OS in GSE31210; OS, overall survival.

Significant differences in overall survival curves between the high- and low-risk classes were seen in the Lung group datasets GSE11969 (matching TCGA type: LUAD and LUSC) and GSE31210 (matching TCGA type: LUAD), and the Female Reproductive group datasets GSE119041 (matching TCGA type: UCEC) and GSE52903 (matching TCGA type: CESC) (Figure 4). Accordingly, the Female Reproductive and Lung groups were internally and externally validated.

### 3.5. Functional Enrichment Analysis

Functional enrichment analysis was performed to identify differences in the pathways between the high- and low-risk classes for the internally and externally validated groups.

**Figure 5:**
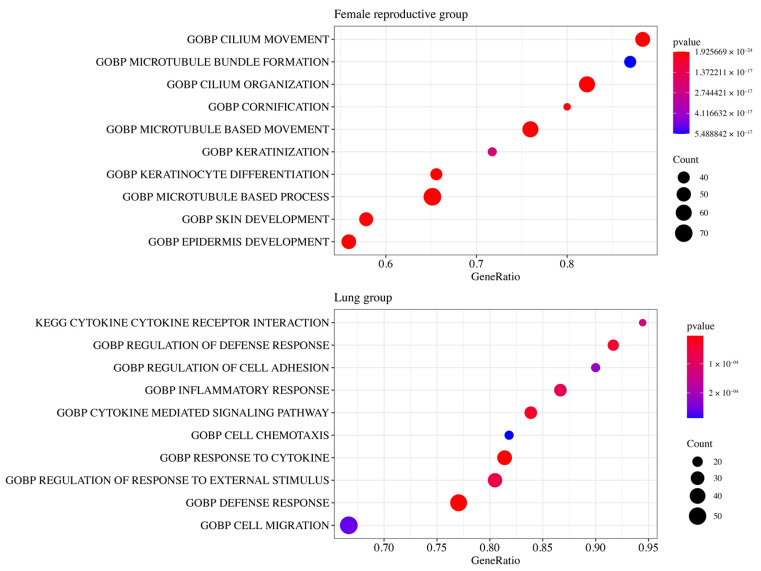
Top 10 enriched terms in the Female Reproductive and Lung groups by functional enrichment analysis. The Female Reproductive group and the Lung group are internally and externally validated groups. DEGs in the Female Reproductive group were enriched in terms related to cilia, while DEGs in the Lung group were enriched in terms related to cytokines.

GSEA shows that 1,638 DEGs between high- and low-risk classes in the Female Reproductive group were enriched in terms related to cilia (‘cilium movement’ and ‘cilium organization’) and terms related to skin (‘skin development’ and ‘epidermis development’) (Figure 5, Table S2).

In the Lung group, 505 DEGs were enriched in terms related to cytokines (‘cytokine’, ‘cytokine receptor interaction’, ‘response to cytokine’, and ‘cytokine mediated signaling pathway’) and defense (‘regulation of defense response’ and ‘defense response’) (Figure 5, Table S3).

### 3.6. Gene Ontology Analysis

The gene ontology (GO) analysis was performed to identify the functions of positive risk coefficient genes.

**Figure 6:**
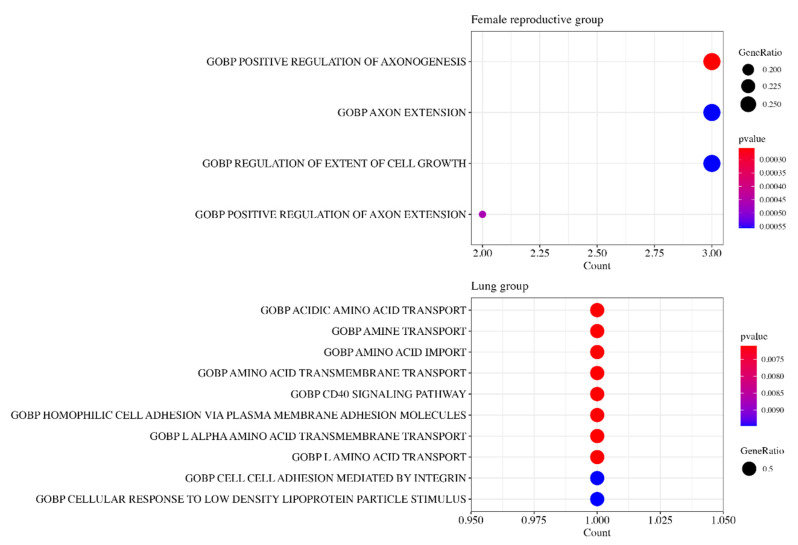
Top 10 enriched terms in the Female Reproductive and Lung groups by functional enrichment analysis. The Female Reproductive group and the Lung group are internally and externally validated groups. The positive risk coefficient genes in the Female Reproductive group were enriched in terms related to axon regulation, while those of the Lung group were related to amino acid transport.

GO analysis shows that 11 positive risk coefficient genes (*BDKRB1*, *CDKN2A*, *FN1*, *ITGA5*, *PFKM*, *SLC45A3*, *TFRC*, *VEGFA*, *WNT3*, *YWHAB*, *YWHAG*) in the Female Reproductive group were enriched in terms related to axon regulation, such as POSITIVE REGULATION OF AXONOGENESIS, AXON EXTENSION, and POSITIVE AXON REGULATION OF AXON EXTENSION (Figure 6).

Two positive risk coefficient genes (*ITGB1*, *LDHA*) in the lung group were related to amino acid transport. Terms such as ACIDIC AMINO ACID TRANSPORT, AMINE TRANSPORT, AMINO ACID IMPORT, etc., were enriched.

## 4. Discussion

Hypoxia induces metabolic and molecular changes in the majority of malignant tumors. The association between the genomic characteristics of hypoxia and aggressive tumor cell phenotypes is well-established [13]. In this study, our principal aim was to investigate the potential role of hypoxia-related genes in cancers that share anatomically close locations. The study revealed that prognoses of the Female Reproductive and Lung groups differed significantly between the low-risk and high-risk group, affected by hypoxia-related genes. Internal validation exhibited suboptimal results within the Liver and Urinary groups, while external validation encountered challenges in the Upper GI and Lower GI groups. The findings implied that the function of hypoxia-related genes in the progression of these cancers might exhibit variability, despite the shared anatomical origins.

CESC and UCEC are representative gynecological cancers. Although these two cancer types have different clinical features, origins, and prognoses, studies have demonstrated that gynecologic cancers share abnormally expressed genes [14]. Two hub genes (*PAMR1* and *SLC24A3*) are potential shared biomarkers for both CESC and UCEC [15]. The expression of *PAMR1* and *SLC24A3* in cancer tissue is downregulated significantly compared to normal tissue. *PAMR1* influences epithelial-to-mesenchymal transition by inhibiting the proliferation, migration, and invasion of cancer cells [16]. *SLC24A3* (also known as *NCKX3*) is involved in the transport of calcium across the cell. Its expression is abundant within the human endometrium at the mRNA and protein levels, especially during menstruation, and it has a role in the reproductive cycle [17]. Another study found that *MAL* overexpression may predict poor prognosis in CESC and UCES [18]. One study reported that *MAL* expression increased in chemo-resistant cancers, and is associated with short overall survival [19]. Expression of *ACTA1*, *MYH7*, and *MYBPC1* may be a potential promotor of gynecological cancer initiation or progression [20]. These genes are regulators of actin and myosin and, as such, changes in actin bundling proteins caused by gene alterations could be correlated with cancer initiation or progression.

In agreement with published studies, the current study identified overlapping molecular findings in gynecological cancer, focusing on the hypoxia gene. We identified 11 positive risk coefficient genes and 7 negative risk coefficient genes. Among them, *CDKN2A* (also known as *P16* gene), *FN1*, and *ITGA5* were identified as tumor markers which predict poor prognosis. *CDKN2A* encodes tumor suppressor protein or tumor immunity [21]. *CDKN2A* methylation has been reported in association with poor prognosis in ovarian cancer [22]. The *FN1* gene is a glycoprotein involved in cell proliferation and migration. The expression of *FN1* is a very poor prognosis marker for various cancer types, including gastric and thyroid cancers [16,23]. Other studies report that expression of *ITGA5* is increased in breast and ovarian cancers compared to normal tissue [24,25]. *ITGA5* is a member of the integrin alpha chain family and combines with *ITGB1* to form integrin α5β1, which has been demonstrated to engage in tumor cell adherence. Progression of gynecological cancer may be promoted by microenvironmental changes in tumor immunity, adherence, and migration caused by alteration of hypoxia-related genes.

We also found that upregulation of certain genes was associated with better prognosis. In the current study, *IL2RB* and *LEF1* were associated with good prognosis in the Female Reproductive group. In contrast to the current findings, high expression of *IL2RB* and *LEF1* has been correlated with poor prognosis. *IL2RB*, as a T-cell-mediated immune system regulating gene, was reported to increase cytotoxic lymphocytes, T-cells, and natural killer cells, leading to immune invasion and tumorigenesis [26]. *LEF1* is essential for T- and B-cell differentiation and its transcription factors are required for self-renewal of leukemic stem cells [27]. Thus, LEF1 is consistently associated with T-cell tumors in the literature.

The GSEA data illustrated that the cilia and epidermis have a vital function in gynecological tumorigenesis. The human endometrium comprises abundant motile cilia, and ciliary defects may play a role in the early stages of tumor development. When the motile cilia decrease, oxidative stress in epithelial cells is exacerbated, which can lead to precursor cancer [14]. The exocervix and vagina are lined with squamous epithelium that form the surface of the skin and hollow organs. If these cells repeatedly suffer from external insult (e.g., repeated infection by human papillomaviruses), cell cycle regulatory tumor suppressor proteins—such as *p53* and *pRB*—are inactivated, allowing epidermal cells in squamous cell carcinoma to abnormally proliferate and dedifferentiate [28]. In gynecological cancers, the functional pathways of epithelial cells are the most important mechanism of cancer progression.

GO analysis showed that axon control is one of the influential factors in the micro-environment of female reproductive cancer. Recently, a study denoted the connection between neuro activity and cancer cell growth [29]. The release of neurotransmitters stimulates the cancer cell and its stromal cell. The process is also facilitated by the growth factor of cancer cells, leading to perineural invasion. Other research also suggested that neural activity such as axonogenesis is also observed in gastric and colon cancer [30,31]. Our findings exhibit a congruent trend with the prior studies, suggesting that neural activity could significantly influence tumor infiltration.

Lung adenocarcinoma and lung squamous cell carcinoma are major cancers which have been investigated during many studies. Although they originate from different cells and have different molecular profiles, some studies have demonstrated common gene pathways. Six genes (*PGK1*, *ENO2*, *GPI*, *PEKP*, *ALDOA*, and *ANGPTL4*) were reported as hypoxia-related genes in lung cancer [32]. These genes function as regulators of oxygen-dependent molecular pathways, leading to increased anaerobic glycolysis. *TTF1*, *KRT7*, *SOX2*, *P63*, and *KRT5* are biomarkers for poor prognosis of lung adenocarcinoma and lung squamous cell carcinoma [33]. Among these genes, *TTF1* was demonstrated to contribute to the maintenance of the function of terminal respiratory unit cells, and is used in immunohistochemistry differential diagnosis of lung cancer [34]. *SOX2*, as a stem cell transcription factor, regulates human somatic cells to pluripotent stem cells. One study revealed that overexpression of *SOX2* amplifies the 3q gene, which is the most common genomic mutation in lung cancer [35].

In this study, *ITGB1* and *LDHA* were poor prognosis markers and *GLS2* was an indolent marker. *ITGB1* has been reported to regulate cancer migration, invasion, and metastasis. Previous studies have shown that knockdown of *ITGB1* reduces breast cancer and colorectal cancer [36,37]. *LDHA* is an enzyme gene involved in creating an acidic microenvironment by effecting the pyruvate cycle. When *LDHA* is overexpressed, epithelial-to-mesenchymal transition is overactivated, which is associated with poor prognosis [38]. The prior research has established that LDHA plays a pivotal role in augmenting glycolytic processes and cellular proliferation, both in in vitro and in vivo [39]. This study illustrated that heightened LDHA expression resulted in amplified glucose uptake and lactate production within cancer cells. In contrast, *GLS-2* has recently been identified as a key gene in the suppression of cancer metastasis via its regulation of glutamine metabolism [40]. It binds to *Rac1-GDP* by inhibiting Rac1 activity, which eventually activates the *p53* tumor suppression gene. The function of the tumor suppressor GLS2 has also undergone previous in vitro studies [41]. Elevating *GLS2* expression within cancer cells has demonstrated an antiproliferative effect, resulting in cell cycle arrest at the G2/M phase. The modification of proteins and other anti-oncogenes through alteration of hypoxia-related genes may determine cancer prognosis.

Functional enrichment analysis indicated that the molecular mechanisms related to cytokine and defense were enriched in lung cancer. Despite controversy that immune proteins alone are not related to cancer risk, cytokine expression in lung cancer has been described in the recent research [42]. Tumor cells yield immunosuppressive cytokines, which are able to avoid attacks by the host’s immune system. The impaired anticancer defense system can also cause the immune system to avoid cancer cells [43]. These mechanisms of immunogenicity in lung cancer may potentially have a crucial function in the regulation of cancer progression.

GO analysis revealed that changes to amino acid profiles accelerate tumor growth in lung cancer. It is known that particular amino acids facilitate the proliferation of cancer cells and their potential role in the regulation of their microenvironment. Among them, tryptophan is a crucial amino acid for immune proliferation through the regulation of T-cells [44]. The change in levels of tryptophan can induce immune escape, leading to promotion of cancer cells. In addition, asparagine, aspartate, and glutamine are known as intracellular and extracellular amino acids which serve to assist in cell integration [45]. When the amino acids are depleted, it cause impairment of protein synthesis and finally lead to unusual apoptosis. All these metabolism shifts can induce a change in the microenvironment, allowing lung cancer to grow well.

The strength of the current study was the analysis of hub genes according to the location of cancer, based on massive data mining. However, there are some limitations. First, as a retrospective study, there is inevitable selection bias. Therefore, we performed external validation with larger sample sizes to overcome the bias. Second, the study did not encompass clinical factors such as sex, age, and stage, which presented a limitation in terms of comprehensiveness. However, it is practically challenging to procure a development set and validation set that encompass the entirety of the clinical factors. The study was deliberately designed to exclusively investigate hypoxia-related genes. Third, as our study focused on the analysis of genes within group categories, it is possible that the sensitivity and precision for individual cancers may be diminished in comparison to other biomarkers. Nonetheless, the significance of our approach lies in its capacity to offer a comprehensive strategy for predicting survival outcomes across a spectrum of diverse cancer types. Therefore, further prospective clinical trials and experimental studies are needed to further validate our findings.

## 5. Conclusions

Our study identified common hypoxia-related genes in female reproductive cancers as well as lung cancers, and detected functional mechanisms to further elucidate the developmental process of cancer. This bioinformatics analysis expands our knowledge of cancer, and may lead to the development of better personalized treatment strategies. Extensive research related to the hypoxia genes is required to predict cancer prognosis through risk stratification.

## Figures and Tables

**Table 1:** Hypoxia-Related Pathway list.

Database	Pathways
KEGG	Pathways in cancer
	Central carbon metabolism in cancer
	PI3K-Akt signaling pathway
	HIF-1 signaling pathway
	VEGF signaling pathway
	TNF signaling pathway
Reactome	Hexose transport
	Signaling by NOTCH1
	MAPK targets/Nuclear events mediated by MAP kinases

KEGG, Kyoto Encyclopedia of Genes.

**Table 2:** List of TCGA Tumor Types in Each Group.

Group	Type (Abbreviation, Number of Samples)
Liver	Liver hepatocellular carcinoma (LIHC, 368) Cholangiocarcinoma (CHOL, 36)
Upper Gastrointestinal	Esophageal carcinoma (ESCA, 185) Stomach adenocarcinoma (STAD, 389)
Lower Gastrointestinal	Colon adenocarcinoma (COAD, 434) Rectum adenocarcinoma (READ, 154)
Female Reproductive	Uterine corpus endometrial carcinoma (UCEC, 531) Cervical squamous cell carcinoma and endocervical adenocarcinoma (CESC, 293)
Urinary	Bladder urothelial carcinoma (BLCA, 406) Kidney renal clear cell carcinoma (KIRC, 532) Kidney renal papillary cell carcinoma (KIRP, 288) Kidney chromophobe (KICH, 65)
Lung	Lung adenocarcinoma (LUAD, 504) Lung squamous cell carcinoma (LUSC, 495)

TCGA, The Cancer Genome Atlas.

**Table 3:** Group Log-Rank Test Results and TCGA Internal Validation Results.

Group	Group-Specific Log-Rank *p*-Value	Type	Type-Specific Log-Rank *p*-Value	Internal Validated
Liver	5.12 × 10^−18^	LIHC	2.92 × 10^−17^	FALSE
		CHOL	6.17 × 10^−2^	
Upper Gastrointestinal	1.39 × 10^−9^	ESCA	1.99 × 10^−2^	TRUE
		STAD	7.88 × 10^−8^	
Lower Gastrointestinal	8.02 × 10^−11^	COAD	5.20 × 10^−9^	TRUE
		READ	2.19 × 10^−2^	
Female Reproductive	9.10 × 10^−16^	UCEC	1.51 × 10^−7^	TRUE
		CESC	5.32 × 10^−4^	
Urinary	9.86 × 10^−47^	BLCA	3.46 × 10^−5^	FALSE
		KIRC	3.75 × 10^−13^	
		KIRP	7.53 × 10^−5^	
		KICH	8.29 × 10^−2^	
Lung	1.51 × 10^−6^	LUAD	7.06 × 10^−5^	TRUE
		LUSC	3.95 × 10^−3^	

LIHC, liver hepatocellular carcinoma; CHOL, cholangiocarcinoma; ESCA, esophageal carcinoma; STAD, stomach adenocarcinoma; CODA, colon adenocarcinoma; READ, rectum adenocarcinoma; UCEC, uterine corpus endometrial carcinoma; CESC, cervical squamous cell carcinoma and endocervical adenocarcinoma; BLCA, bladder urothelial carcinoma; KIRC, kidney renal clear cell carcinoma; KIRP, kidney renal papillary cell carcinoma; KICH, kidney chromophobe; LUAD, lung adenocarcinoma; LUSC, lung squamous cell carcinoma.

**Table 4:** List of Group-Specific Hypoxia Risk Genes.

Group	Positive Risk Coefficient	Negative Risk Coefficient
Liver	*BIRC5*, *BIRC8*, *CUL2*, *EIF4E*, *EPO*, *G6PD*, *GNA12*, *HDAC1*, *HDAC2*, *HSP90AA1*, *IFNA13*, *IL8*, *LDHA*, *MAPK7*, *NUP155*, *PGF*, *PPP2R5B*, *RHEB*, *SLC1A5*, *SLC2A1*, *SPP1*, *YWHAB*	*CCNA1*, *CNTN1*, *FLT3*, *G6PC2*, *GHR*, *HES5*, *IFNA2*, *ITGB7*, *NTRK1*, *PFKL*, *TNF*, *TP53*, *WNT1*
Upper Gastrointestinal	*APH1B*, *SERPINE1*, *SLC2A3*, *SOCS3*, *TF*	*DAB2IP*, *MKNK2*
Lower Gastrointestinal	*APC2*, *ENO3*, *HEYL*, *TIMP1*, *WNT10B*	*CTNNA1*, *MAPKAPK3*, *TMEM48*
Female Reproductive	*BDKRB1*, *CDKN2A*, *FN1*, *ITGA5*, *PFKM*, *SLC45A3*, *TFRC*, *VEGFA*, *WNT3*, *YWHAB*, *YWHAG*	*CD19*, *IL2RB*, *JMJD7-PLA2G4B*, *LEF1*, *MDM2*, *RBPJ*
Urinary	*BIRC5*, *CCNE2*, *COL6A3*, *DVL3*, *EIF4EBP1*, *FGF5*, *GLI2*, *PPP2R2C*, *SLC7A5*, *THBS3*	*DAB2IP*, *ITGB7*
Lung	*ITGB1*, *LDHA*	*GLS2*

**Table 5:** Datasets Used For External Validation and Log-Rank Test Results.

Group	Matched TCGA Type	GEO Accession Number
Upper Gastrointestinal	ESCA	GSE72873
	STAD	GSE15459 *
Lower Gastrointestinal	COAD READ	GSE41258, GSE17538, GSE72970, GSE17537, GSE17536
Female Reproductive	UCEC	GSE119041 *
	CESC	GSE52903 *
Urinary	BLCA	GSE31684, GSE13507, GSE19423
	KIRC	GSE29609
Lung	LUAD/SC	GSE11969 *, GSE37745
	LUAD	GSE31210 *, GSE30219, GSE50081, GSE29014

* *p*-value < 0.05; ESCA, esophageal carcinoma esophageal carcinoma; STAD, stomach adenocarcinoma; CODA, colon adenocarcinoma; READ, rectum adenocarcinoma; UCEC, uterine corpus endometrial carcinoma; CESC, cervical squamous cell carcinoma and endocervical adenocarcinoma; BLCA, bladder urothelial carcinoma; KIRC, kidney renal clear cell carcinoma; LUAD, lung adenocarcinoma; LUSC, lung squamous cell carcinoma.

## Data Availability

The web links of the related public datasets or databases are as follows: TCGA: https://tcga-data.nci.nih.gov/tcga/; https://gdc.cancer.gov/about-data/publications/pancanatlas; GEO: https://www.ncbi.nlm.nih.gov/geo/.

## Supplementary Materials

The following supporting information can be downloaded at: https://www.mdpi.com/article/10.3390/medicines11010002/s1. Table S1: Datasets for hypoxia-related gene list, Table S2: Datasets Used for External Validation and Log-Rank Test Results, Table S3: Gene set enrichment analysis results for DEGs in the Female Reproductive group (Top 10), Table S4: Gene set enrichment analysis results for DEGs in the Lung group (Top 10).

## References

[1] Barron C.C., Bilan P.J., Tsakiridis T., Tsiani E. (2016). Facilitative glucose transporters: Implications for cancer detection, prognosis and treatment. Metab. Clin. Exp..

[2] D’Ignazio L., Batie M., Rocha S. (2017). Hypoxia and Inflammation in Cancer, Focus on HIF and NF-kappaB. Biomedicines.

[3] Chai Y.J., Yi J.W., Oh S.W., Kim Y.A., Yi K.H., Kim J.H., Lee K.E. (2017). Upregulation of SLC2 (GLUT) family genes is related to poor survival outcomes in papillary thyroid carcinoma: Analysis of data from the Cancer Genome Atlas. Surgery.

[4] Gan L., Meng J., Xu M., Liu M., Qi Y., Tan C., Wang Y., Zhang P., Weng W., Sheng W. (2018). Extracellular matrix protein 1 promotes cell metastasis and glucose metabolism by inducing integrin β4/FAK/SOX2/HIF-1α signaling pathway in gastric cancer. Oncogene.

[5] Zhang Z., Wang A., Tang X., Chen Y., Tang E., Jiang H. (2020). Comparative mutational analysis of distal colon cancer with rectal cancer. Oncol. Lett..

[6] Yerukala Sathipati S., Tsai M.J., Carter T., Allaire P., Shukla S.K., Beheshti A., Ho S.Y. (2022). Survival estimation in patients with stomach and esophageal carcinoma using miRNA expression profiles. Comput. Struct. Biotechnol. J..

[7] Salem M.E., Puccini A., Xiu J., Raghavan D., Lenz H.-J., Korn W.M., Shields A.F., Philip P.A., Marshall J.L., Goldberg R.M. (2018). Comparative Molecular Analyses of Esophageal Squamous Cell Carcinoma, Esophageal Adenocarcinoma, and Gastric Adenocarcinoma. Oncologist.

[8] Zhang Z., Furge K.A., Yang X.J., Teh B.T., Hansel D.E. (2010). Comparative gene expression profiling analysis of urothelial carcinoma of the renal pelvis and bladder. BMC Med. Genom..

[9] Weinstein J.N., Collisson E.A., Mills G.B., Shaw K.R.M., Ozenberger B.A., Ellrott K., Sander C., Stuart J.M., Chang K., Creighton C.J. (2013). The cancer genome atlas pan-cancer analysis project. Nat. Genet..

[10] Kanehisa M. (2019). Toward understanding the origin and evolution of cellular organisms. Protein Sci..

[11] Friedman J., Hastie T., Tibshirani R. (2010). Regularization paths for generalized linear models via coordinate descent. J. Stat. Softw..

[12] Wu T., Hu E., Xu S., Chen M., Guo P., Dai Z., Feng T., Zhou L., Tang W., Zhan L. (2021). ClusterProfiler 4.0: A universal enrichment tool for interpreting omics data. Innovation.

[13] López-Cortés A., Guevara-Ramírez P., Guerrero S., Ortiz-Prado E., García-Cárdenas J.M., Zambrano A.K., Armendáriz-Castillo I., Pérez-Villa A., Yumiceba V., Varela N. (2020). Metastatic signaling of hypoxia-related genes across TCGA Pan-Cancer types. bioRxiv.

[14] Guo Y., Liu J., Luo J., You X., Weng H., Wang M.M., Ouyang T., Li X., Liao X., Wang M.M. (2020). Molecular Profiling Reveals Common and Specific Development Processes in Different Types of Gynecologic Cancers. Front. Oncol..

[15] Yu S.H., Cai J.H., Chen D.L., Liao S.H., Lin Y.Z., Chung Y.T., Tsai J.J.P., Wang C.C.N. (2021). Lasso and bioinformatics analysis in the identification of key genes for prognostic genes of gynecologic cancer. J. Pers. Med..

[16] Sun Y., Ye Y., Wang Z., He Y., Li Y., Mao H., Zhao C. (2020). High expression of fibronectin 1 indicates poor prognosis in gastric cancer. Oncol. Lett..

[17] Jalloul A.H., Szerencsei R.T., Schnetkamp P.P.M. (2016). Cation dependencies and turnover rates of the human K^+^-dependent Na^+^-Ca^2+^ exchangers NCKX1, NCKX2, NCKX3 and NCKX4. Cell Calcium.

[18] Liu J., Feng M., Li S., Nie S., Wang H., Wu S., Qiu J., Zhang J., Cheng W. (2020). Identification of molecular markers associated with the progression and prognosis of endometrial cancer: A bioinformatic study. Cancer Cell Int..

[19] Zanotti L., Romani C., Tassone L., Todeschini P., Tassi R.A., Bandiera E., Damia G., Ricci F., Ardighieri L., Calza S. (2017). MAL gene overexpression as a marker of high-grade serous ovarian carcinoma stem-like cells that predicts chemoresistance and poor prognosis. BMC Cancer.

[20] Jha A., Khan Y., Mehdi M., Karim M.R., Mehmood Q., Zappa A., Rebholz-Schuhmann D., Sahay R. (2017). Towards precision medicine: Discovering novel gynecological cancer biomarkers and pathways using linked data. J. Biomed. Semant..

[21] Chen Z., Guo Y., Zhao D., Zou Q., Yu F., Zhang L., Xu L. (2021). Comprehensive Analysis Revealed that CDKN2A is a Biomarker for Immune Infiltrates in Multiple Cancers. Front. Cell Dev. Biol..

[22] Xia L., Zhang W., Gao L. (2019). Clinical and prognostic effects of CDKN2A, CDKN2B and CDH13 promoter methylation in ovarian cancer: A study using meta-analysis and TCGA data. Biomarkers.

[23] Chen C., Shen Z. (2022). FN1 promotes thyroid carcinoma cell proliferation and metastasis by activating the NF-kappaB pathway. Protein Pept. Lett..

[24] Xiao Y., Li Y., Tao H., Humphries B., Li A., Jiang Y., Yang C., Luo R., Wang Z. (2018). Integrin α5 down-regulation by miR-205 suppresses triple negative breast cancer stemness and metastasis by inhibiting the Src/Vav2/Rac1 pathway. Cancer Lett..

[25] Gong C., Yang Z., Wu F., Han L., Liu Y., Gong W. (2016). MIR 17 inhibits ovarian cancer cell peritoneal metastasis by targeting ITGA5 and ITGB1. Oncol. Rep..

[26] Valle-Mendiola A., Gutiérrez-Hoya A., Lagunas-Cruz M.D.C., Weiss-Steider B., Soto-Cruz I. (2016). Pleiotropic Effects of IL-2 on Cancer: Its Role in Cervical Cancer. Mediators Inflamm..

[27] Yu S., Li F., Xing S., Zhao T., Peng W., Xue H.H. (2016). Hematopoietic and leukemic stem cells have distinct dependence on Tcf1 and Lef1 transcription factors. J. Biol. Chem..

[28] Dueñas-González A., Lizano M., Candelaria M., Cetina L., Arce C., Cervera E. (2005). Epigenetics of cervical cancer. An overview and therapeutic perspectives. Mol. Cancer.

[29] Jobling P., Pundavela J., Oliveira S.M.R., Roselli S., Walker M.M., Hondermarck H. (2015). Nerve-cancer cell cross-talk: A novel promoter of tumor progression. Cancer Res..

[30] Zhao C.M., Hayakawa Y., Kodama Y., Muthupalani S., Westphalen C.B., Andersen G.T., Flatberg A., Johannessen H., Friedman R.A., Renz B.W. (2014). Denervation suppresses gastric tumorigenesis. Sci. Transl. Med..

[31] Albo D., Akay C.L., Marshall C.L., Wilks J.A., Verstovsek G., Liu H., Agarwal N., Berger D.H., Ayala G.E. (2011). Neurogenesis in colorectal cancer is a marker of aggressive tumor behavior and poor outcomes. Cancer.

[32] Chen Y.L., Zhang Y., Wang J., Chen N., Fang W., Zhong J., Liu Y., Qin R., Yu X., Sun Z. (2019). A 17 gene panel for non-small-cell lung cancer prognosis identified through integrative epigenomic-transcriptomic analyses of hypoxia-induced epithelial–mesenchymal transition. Mol. Oncol..

[33] Sun F., Yang X., Jin Y., Chen L., Wang L., Shi M., Zhan C., Shi Y., Wang Q. (2017). Bioinformatics analyses of the differences between lung adenocarcinoma and squamous cell carcinoma using the Cancer Genome Atlas expression data. Mol. Med. Rep..

[34] Kim J.H., Kim H.S., Kim B.J., Han B., Choi D.R., Kwon J.H. (2018). Prognostic impact of TTF-1 expression in non-squamous non-small-cell lung cancer: A meta-analysis. J. Cancer.

[35] Karachaliou N., Rosell R., Viteri S. (2013). The role of SOX2 in small cell lung cancer, lung adenocarcinoma and squamous cell carcinoma of the lung. Transl. Lung Cancer Res..

[36] Klahan S., Huang W.C., Chang C.M., Wong H.S., Huang C.C., Wu M.S., Lin Y.C., Lu H.F., Hou M.F., Chang W.C. (2016). Gene expression profiling combined with functional analysis identify integrin beta1 (ITGB1) as a potential prognosis biomarker in triple negative breast cancer. Pharmacol. Res..

[37] Zhang J., Liu K., Peng P., Li S., Ye Z., Su Y., Liu S., Qin M., Huang J. (2019). Upregulation of nectin-4 is associated with ITGB1 and vasculogenic mimicry and may serve as a predictor of poor prognosis in colorectal cancer. Oncol. Lett..

[38] Hou X., Shi X., Zhang W., Li D., Hu L., Yang J., Zhao J., Wei S., Wei X., Ruan X. (2021). LDHA induces EMT gene transcription and regulates autophagy to promote the metastasis and tumorigenesis of papillary thyroid carcinoma. Cell Death Dis..

[39] Xiao X., Huang X., Ye F., Chen B., Song C., Wen J., Zhang Z., Zheng G., Tang H., Xie X. (2016). The miR-34a-LDHA axis regulates glucose metabolism and tumor growth in breast cancer. Sci. Rep..

[40] Zhang C., Liu J., Zhao Y., Yue X., Zhu Y., Wang X., Wu H., Blanco F., Li S., Bhanot G. (2016). Glutaminase 2 is a novel negative regulator of small GTPase Rac1 and mediates p53 function in suppressing metastasis. Elife.

[41] Lopez de la Oliva A.R., Campos-Sandoval J.A., Gomez-Garcia M.C., Cardona C., Martin-Rufian M., Sialana F.J., Castilla L., Bae N., Lobo C., Penalver A. (2020). Nuclear Translocation of Glutaminase GLS2 in Human Cancer Cells Associates with Proliferation Arrest and Differentiation. Sci. Rep..

[42] Peng W.J., He Q., Yang J.X., Wang B.X., Lu M.M., Wang S., Wang J. (2012). Meta-analysis of association between cytokine gene polymorphisms and lung cancer risk. Mol. Biol. Rep..

[43] Domagala-Kulawik J. (2015). The role of the immune system in non-small cell lung carcinoma and potential for therapeutic intervention. Transl. Lung Cancer Res..

[44] Li C., Zhao H. (2021). Tryptophan and Its Metabolites in Lung Cancer: Basic Functions and Clinical Significance. Front. Oncol..

[45] Fahrmann J.F., Vykoukal J.V., Ostrin E.J. (2020). Amino Acid Oncometabolism and Immunomodulation of the Tumor Microenvironment in Lung Cancer. Front. Oncol..

