# Supplementary material for: Expression Profiles of Hypoxia-Related Genes of Cancers Originating from Anatomically Similar Locations Using TCGA Database Analysis
Source: Medicines (Basel). 2023 Dec 31;11(1):2. doi: 10.3390/medicines11010002 (PMC10819830; doi:10.3390/medicines11010002)
Supplement: Supplementary file 1 [file medicines-11-00002-s001.zip › medicines-2425506-supplementary.pdf]

Table S1. Datasets for hypoxia-related gene list

Original	Approved symbol	Gene group
HIF1A	HIF1A	
HIF1B	ARNT	
HIF2A (EPAS)	EPAS1	
HIF2B	ARNT2	
HI3A	HIF3A	
EPO	EPO	
EPOR	EPOR	
G6PC	G6PC	
G6PC2	G6PC2	
G6PC3	G6PC3	
G6PD	G6PD	
HK1	HK1	
HK2	HK2	
HK3	HK3	
HKDC1	HKDC1	
VEGF	VEGFA	VEGF family
	VEGFB	VEGF family
	VEGFC	VEGF family
	VEGFD	VEGF family
	PGF	
PDGF	PDGFA	PDGF family
	PDGFB	PDGF family
	PDGFC	PDGF family
	PDGFD	PDGF family
PAI1	SERPINE1	
SCF	KITLG	
BMP4	BMP4	
CA IX	CA9	
myc	MYC	
p53	TP53	
VHL	VHL	
TGF-B	TGFB1	
MMP-1	MMP1	MMP family
MMP-2	MMP2	MMP family
MMP-3	MMP3	MMP family

MMP-4	ILF3	MMP family
MMP-5	MMP24	MMP family
MMP-6	MMP25	MMP family
MMP-7	MMP7	MMP family
MMP-8	MMP8	MMP family
MMP-9	MMP9	MMP family
	MMP10	MMP family
	MMP11	MMP family
	MMP12	MMP family
	MMP13	MMP family
	MMP14	MMP family
	MMP15	MMP family
	MMP16	MMP family
	MMP17	MMP family
	MMP19	MMP family
	MMP20	MMP family
	MMP21	MMP family
	MMP23A	MMP family
	MMP23B	MMP family
	MMP26	MMP family
	MMP27	MMP family
	MMP28	MMP family
PI3K	PIK3R1	PI3K family
	PIK3R2	PI3K family
	PIK3R3	PI3K family
	PIK3R6	PI3K family
	PIK3R5	PI3K family
MAPK	MAPK1	MAPK family
	MAPK3	MAPK family
	MAPK6	MAPK family
	MAPK4	MAPK family
	MAPK14	MAPK family
	MAPK12	MAPK family
	MAPK8	MAPK family
	MAPK11	MAPK family
	MAPK10	MAPK family
	MAPK9	MAPK family
	MAPK13	MAPK family
	MAPK7	MAPK family

	MAPK15	MAPK family
BRAF	BRAF	
NRAS	NRAS	
HRAS	HRAS	
KRAS	KRAS	
PHD3	EGLN3	
HDAC-1	HDAC1	
HDAC-2	HDAC2	
HDAC-3	HDAC3	
HDAC-4	HDAC4	
HDAC-5	HDAC5	
HDAC-6	HDAC6	
HDAC-7	HDAC7	
HDAC-8	HDAC8	
HDAC-9	HDAC9	
HDAC-10	HDAC10	
HDAC-11	HDAC11	
SLC2A1	SLC2A1	
SLC2A2	SLC2A2	
SLC2A3	SLC2A3	
SLC2A4	SLC2A4	
SLC2A5	SLC2A5	
SLC2A6	SLC2A6	
SLC2A7	SLC2A7	
SLC2A8	SLC2A8	
SLC2A9	SLC2A9	
SLC2A10	SLC2A10	
SLC2A11	SLC2A11	
SLC2A12	SLC2A12	
SLC2A13	SLC2A13	
SLC2A14	SLC2A14	

Table S2. Datasets Used for External Validation and Log Rank Test Results

GEO	Group	log rank p-value	Matched TCGA Type
GSE72873	Upper GI	4.8.E-01	ESCA
GSE15459	Upper GI	3.6.E-02	STAD

GSE17536	Lower GI	9.8.E-01	COAD
GSE17537	Lower GI	9.5.E-01	COAD
GSE17538	Lower GI	7.3.E-01	COAD
GSE41258	Lower GI	1.2.E-01	COAD
GSE72970	Lower GI	7.9.E-01	COAD
GSE119041	Female reproductive	1.6.E-02	UCEC
GSE52903	Female reproductive	5.4.E-03	CESC
GSE13507	Genitourinary	2.6.E-01	BLCA
GSE19423	Genitourinary	5.5.E-01	BLCA
GSE31684	Genitourinary	7.7.E-02	BLCA
GSE29609	Genitourinary	5.1.E-01	KIRC
GSE11969	Lung	5.0.E-02	LUAD/SC
GSE29013	Lung	6.2.E-01	LUAD
GSE30219	Lung	4.8.E-01	LUAD
GSE31210	Lung	1.9.E-04	LUAD
GSE37745	Lung	3.8.E-01	LUAD/SC
GSE50081	Lung	5.0.E-01	LUAD

ESCA, Esophageal carcinoma esophageal carcinoma; STAD, Stomach adenocarcinoma; CODA, Colon adenocarcinoma; UCEC, Uterine corpus endometrial carcinoma; CESC, Cervical squamous cell carcinoma and endocervical adenocarcinoma; BLCA, Bladder urothelial carcinoma; KIRC, Kidney renal clear cell carcinoma; LUAD, Lung adenocarcinoma; LUSC, Lung squamous cell carcinoma

Table S3. Gene set enrichment analysis results for DEGs in the Female reproductive group (Top 10)

Description	Enrichment Score	p.adjust
Microtubule based process	-0.5792769	1.64E-21
Cilium organization	-0.6485697	4.60E-20
Cilium movement	-0.6847378	4.57E-19
Epidermis development	0.55117449	6.71E-18
Keratinocyte defferentiation	0.6326259	2.28E-17
Cornification	0.72642571	5.68E-17
Microtubule based movement	-0.59232	5.68E-17
Skin development	0.56568153	8.67E-17
Keratinization	0.67393106	2.14E-15
Microtubule based formation	-0.6737431	4.68E-15

Table S4. Gene set enrichment analysis results for DEGs in the Lung group (Top 10)

Description	Enrichment Score	p.adjust
Response to cytokine	0.46734305	0.00051058
Defense response	0.41871499	0.00051058
Cytokine mediated signaling pathway	0.49224008	0.00267439
Regulation of defense response	0.53735122	0.00267439
Regulation of response of external stimulation	0.43903768	0.00346152
Inflammatory response	0.4871903	0.00352808
Cytokine cytokine receptor interation	0.57279785	0.00510792
Regulation of cell adhesion	0.53904955	0.00757874
Cell migration	0.3254014	0.00793285
Cell chemotasix	0.51092724	0.00793285
